# Qualitative analysis of UK women’s attitudes to calorie-based alcohol marketing and alcohol calorie labelling

**DOI:** 10.1093/heapro/daae006

**Published:** 2024-02-21

**Authors:** Amanda M Atkinson, Beth R Meadows, Erin Hobin, Lana Mae Vanderlee, Harry Sumnall

**Affiliations:** Public Health Institute, Liverpool John Moores University, 3rd Floor Exchange Station, Tithebarn Street, Liverpool L2 2QP, UK; School of Health and Life Sciences, Glasgow Caledonian University, Cowcaddens Road, Glasgow, Scotland G4 0BA, UK; Public Health Ontario, 480 University Ave #300, Toronto, ON M5G 1V2, Canada; School of Nutrition, Université Laval, 2425 rue de l’Agriculture, Québec, QC G1V 0A6, Canada; Public Health Institute, Liverpool John Moores University, 3rd Floor Exchange Station, Tithebarn Street, Liverpool L2 2QP, UK

**Keywords:** alcohol, women, marketing, calorie information, drinking

## Abstract

Mandatory standardized nutritional information on alcoholic drinks such as energy, or calorie labelling, is a population-level public health measure aimed at addressing obesity and alcohol consumption. In the UK, such measures are not a statutory requirement, but some alcohol brands do include references to calories on their products and in their marketing materials, as a marketing strategy to encourage sales and consumption. This article presents findings of semi-structured individual (*N* = 43) and group (*N* = 9) interviews with 78 women living in the UK that aimed to gain insight into their attitudes towards calorie-based alcohol brand marketing, and alcohol calorie labelling (ACL) as a health policy. Three themes are presented that outline how women rejected calorie marketing and labelling; the potential positive and unintended impact on alcohol consumption and dietary/eating practices; and how views on calorie labelling were intertwined with women’s attitudes towards marketing that draws on calorie messaging. A feminist anti-diet discourse, as well as a discourse of pleasure through alcohol consumption, was at play in women’s accounts, which may limit the intended aims of ACLs. It is concluded that ACLs should be considered within the wider commercial context of alcohol marketing that draws on calories to promote sales and consumption, consideration of the gendered factors that may lead some to reject ACLs as a health policy, and the potential for unintended consequences.

**Contribution to Health Promotion**

- This research contributes to understanding the commercial context of calorie-based alcohol brand promotion, in which standardized alcohol calorie labelling (ACLs) as a form of health promotion is placed.
- Alcohol products marketed as low-calorie and ACLs may appeal to some women who already employ strategies to reduce calorie intake from alcohol consumption.
- However, many women were critical of calorie marketing and ACLs.
- They rejected both through a feminist discourse that critiqued calorie references for undermining body positivity and through a discourse of pleasure that prioritized the fun associated with drinking.

## BACKGROUND

Mandatory nutritional information on alcoholic drinks such as energy, or calorie labelling, is a population-level public health measure aimed at addressing obesity and alcohol consumption (Maynard *et al*., 2018; Robinson *et al*., 2021a). It is based on the premise that providing standardized information is a consumer right that will enhance awareness and knowledge, support consumers in making ‘informed choices’ on food and alcohol intake, and in turn improve health through encouraging the selection of lower calorie options, reduced alcohol consumption and changes in diet and exercise (Clarke *et al*, 2021a; Robinson *et al*., 2021b).

It has been estimated that 9% of UK drinking adults’ daily calorie intake comes from alcohol (Bates *et al*., 2011), yet consumer knowledge on the calories in alcoholic drinks is poor (Gibson and Shirreffs, 2013; AHA, 2021b; Dimova and Mitchell, 2021; Robinson *et al*., 2021a). Representative polls of UK adults (*N* = 1633) and young people (*N* = 3388), have found that around 60% supported mandating standardized alcohol calorie labelling (ACLs) (AHA, 2021a; IAS, 2021), and a survey of 450 UK adults found that 81% agreed that calorie information on alcoholic drinks is a ‘good idea’ (Maynard *et al*., 2018). Whilst there is a lack of qualitative research on consumer attitudes to ACLs, focus group research (RSPH, 2018) with 24 adult drinkers in the North and South of England, found that calorie information was placed in the top three priorities for alcohol labelling, alongside ABV and units. This suggests strong support for ACLs, which could offer a way to address gaps in consumer knowledge to assist decision making. However, there is a limited evidence based on the effectiveness of ACLs on intentions to purchase, patterns of alcohol consumption and long-term impact on health (Gibson and Shirreffs, 2013; Clarke *et al*., 2021b; Dimova and Mitchell, 2021; Robinson *et al*., 2021a).

In the UK, unlike food items, uniform calorie labelling on alcoholic drinks is not a statutory requirement. The Portman Group (2022a), the UK industry body that regulates alcohol labelling, packaging and promotion within a self-regulatory framework, provides producers with advice on how to incorporate calorie information if they so wish (stating ‘*where provided [it] must be displayed per 100ml*’) and various European trade bodies (e.g. Brewers of Europe, 2019; Spirits Europe, 2019) have signed agreements to incorporate calorie information on products. In the last decade there appears to have been an increase in the energy labelling of alcoholic products sold in UK supermarkets (Robinson *et al*., 2021b), yet voluntary pledges and industry guidance are limited in their effectiveness and have been discussed as a tactic used by industry to offset mandatory requirements (Robinson *et al*., 2021b). A representative study of main UK supermarkets found that, despite producers and retailers committing to providing calorie information (i.e. ‘*simple and consistent information as appropriate in the off-trade (supermarkets and off-licences)*’) as part of the 2011 Public Health Responsibility Deal, calorie information was not routinely provided on supermarket websites or on product labels, following the commitment (Petticrew *et al*., 2017). A Portman Group report (2022b) presented findings of an analysis of 400 alcohol products on display in UK supermarkets between July and August 2021, stating that 47% displayed calorie information on labels, ‘*most often*’ depicting kilojoules (kj)/kilocalories (kcal) per 100 ml, in line with existing food labelling regulations. The report does not however state whether calorie information was presented in a consistent and uniform format on the back of containers.

Public health bodies (e.g. AHA, 2020; WHO, 2022) have called for the labelling of nutritional information on alcoholic drinks including calories to be made a legal requirement, and as one strategy that can help reduce alcohol harm and obesity. The European Commission is in the process of proposing mandatory nutrition and ingredient labelling and health warnings as part of Europe’s Beating Cancer Plan, and in Ireland, energy labelling is due to come into force as part of the 2018 Public Health (Alcohol) Act (IAS, 2021). In 2020, the UK Government announced an intention to consult on the mandatory calorie labelling of alcoholic drinks as part of their obesity strategy (DCSH, 2020). As Robinson *et al*. (2023) assert, for ACLs to reduce obesity and alcohol use, it must directly change population-level energy balance by causing individuals to change their behaviour (e.g. choosing lower energy drink options or increasing physical activity), or indirectly change behaviour by altering alcohol industry practices including the reformulation and development of alcoholic drinks that have lower energy content (e.g. through reductions to serving sizes and ABV).

While calorie labelling is advocated as a potentially effective health policy, research has found that many brands do currently utilize calorie information. However, this is not in the form of standardized nutritional information, but as a marketing strategy to encourage sales and consumption. In an analysis (Atkinson *et al.*, 2022a) of alcohol marketing posts on social media, brands were found to target women with calorie messaging, such as ‘*Skinny*’ recipes and products, and slogans such as ‘*only 70 calories per can*’ (Atkinson *et al*., 2021). This includes Skinny Prosecco (Skinny Prosecco, 2022), a product arguably targeted at women, which is promoted as a ‘*guilt-free*’ option ‘*with only 82.6 calories per glass*’. Similarly, a recent (Atkinson *et al.*, 2022b) analysis of alcohol content in women’s magazines, found that associations between alcohol and weight were common, and that magazines regularly promoted the sale and use of alcoholic products to women through reference to calories, and dieting. Products that display calorie information have also been referred to as ‘better-for-you’ and ‘health-oriented’ products (Keric *et al*., 2022) that can create a ‘health halo’ by misleading consumers of health benefits. This may lead to the misperception of these products are ‘healthier’ options, whilst detracting from the harms associated with alcohol use (Haynes *et al*., 2022). For example, a randomized online experiment with Australian women (*N* = 501) (Cao *et al.*, 2023) found that those who were exposed to alcohol products with low sugar claims rated them as healthier, less harmful to health, (incorrectly) lower in alcohol and as aids in weight management and a healthy diet, compared to the same products without claims. Thus, some brands incorporate calorie information in their packaging (i.e. on the front of the package), marketing promotions (i.e. calorie-based slogans) and brand identity (e.g. ‘*Skinny beer*’) as a strategy designed to instigate the sale and use of alcoholic drinks, rather than for health promotion.

Lack of consumer awareness of the energy content of alcoholic drinks alongside evidence of public support for ACLs, suggests there may be a need for standardized calorie labelling (Robinson *et al*., 2021b). There are obvious differences between the use of calories as a marketing tactic to encourage sales and use, and the inclusion of uniform calorie labelling as a health policy (e.g. purpose, format, location, consistency across products). However, calorie-based marketing is a real-world strategy found to predominantly target women, and as such, women’s attitudes towards to it are likely to influence, and shed light on, consumer perceptions of standardized calorie labelling. The effectiveness of ACLs will also be influenced by existing consumer attitudes towards the use of calories as a marketing strategy, the acceptably of ACLs, and perceived intentions and impact. There is a lack of qualitative research on the appeal of such marketing, and how it is considered in relation to ACLs. Research is also needed with women as they tend to be more weight and health conscious, and this may make them more susceptible to calorie information (Robinson *et al*., 2021b). However, there has been a shift in the sociocultural climate in which the female body is constructed and positioned in recent years. Media and marketing culture has long promoted slimness and thinness as beauty ideals that women are pressured to adhere to, in ways that promote restrained consumption and body dissatisfaction (van den Berg *et al.*, 2007; Mooney *et al.*, 2009; López-Guimerà *et al.*, 2010; Swiatkowski, 2016). More recently, an anti-diet and body positivity movement has emerged in pop-cultural discourse, and this aims to widen the definition of female beauty and the acceptably of a variety of body shapes and sizes (Cohen *et al*., 2020; Griffin *et al*., 2022). Such discourse may influence the way women perceive and engage with calorie-based marketing and calorie labelling. To address gaps in research, this article presents findings of qualitative individual and group interviews with women in the UK, which aimed to gain insight into their attitudes towards the use of calorie messaging as a marketing strategy by alcohol brands, and calorie labelling as a public health policy.

## METHODS

Semi-structured individual (*N* = 43) and group (*N* = 9) interviews with 78 women living in the UK were conducted to gain insight into their attitudes towards calorie-based alcohol brand marketing, and ACLs. The research formed one element of a wider Economic and Social Research Council (ES/TOO7443) funded study exploring the nature, influence, creation and regulation of alcohol marketing in the age of contemporary feminism. To encourage participation, openness and trustworthiness, participants were given a choice of taking part in an individual or group interview. Those taking part in a group interview did so with their friends. The sample size and diversity of participants with regards to demographics allowed us to capture differences and similarities in opinions and experiences. In the interviews, participants were asked whether they considered calories when consuming alcohol, how they felt about calories being used in the marketing of alcohol products and whether calorie information would influence their own and others purchasing and consumption. Photo elicitation was used to gain views on examples of marketing content referring to calories extracted from a systematic search and analysis of alcohol brand content on social media (Atkinson *et al.*, 2022a). Once gaining their views on calorie-based marketing, they were asked to express their opinions on mandatory calorie labelling (i.e. standardized information on the back of all products) as a health policy to reduce alcohol use and obesity, and how they felt it would influence their own and other consumers purchasing and alcohol consumption.

Participants were recruited through researcher networks (i.e. friends, family, colleagues), snowballing, flyers in public spaces such as bars, cafes and workplaces, a project Instagram page, advertising to university and college students and societies, and community groups. The majority drank alcohol, but 12% (*N* = 16) were currently sober due to self-defined problematic use. The majority resided in one city in the North of England, but the 12% (*N* = 14) who did not consume alcohol lived in other areas of the UK (i.e. Scotland, Northern Ireland, North East and South East England). Ages ranged between 19 and 50 (one was in their 40s, and one 50, and most were in their 20s). The majority (83%, n = 65) were White British (Black British 14% (*N* = 11); British Asian 1% (*N* = 1); British Mixed 1% (*N* = 1)), and heterosexual (89%, *N* = 69; LGBTQ+ 12%, (*N* = 9)). Interviews were conducted and recorded online by the first and second authors via Microsoft Teams. Automatic transcriptions were used as a guide and recordings were re-reviewed to amend the transcription and provide a verbatim account. A thematic analysis, led by the first author with input from the second (code checking, discussion of coding), was conducted in NVivo to develop patterns, themes and sub-themes through numerous close readings of the transcripts and discussion with other authors (Braun and Clarke, 2006). A broad list of areas to be explored was devised in advance (e.g. negative and positive attitudes, drinking intentions) and was updated and refined during the analysis as themes arose inductively. An integrative approach to thematic analysis was used, with new themes identified within the interview data, being included in the coding frame and then applied to previously and yet-to-be-coded transcripts.

The identities of the researchers that conducted the interviews as white women aged 24 and 38, may have influenced the discussions. On reflection, we felt that our gender allowed us to build rapport with all women, and they appeared eager to share their views and experiences openly and enthusiastically. The status of the researchers as consumers of alcohol may have also influenced discussions. For example, at times, non-drinking participants appeared to sell and promote sobriety to the researchers; however, like those who drank alcohol, they were eager to express their views. To ensure that our positionality as feminist scholars did not bias responses (e.g. a reluctance among women to present opposition to feminist thinking), we did not overtly present our positions and when asking women to clarify whether they identified as feminist themselves, we asked in a non-judgemental and neutral manner. Ethical approval was granted by the University Research Ethics Committee and informed verbal consent was gained from each participant.

## RESULTS

### ‘*They want us to be insecure so they can make money*’: Rejecting calorie marketing and labelling through a feminist discourse

Most women interpreted the inclusion of calories in brand marketing and the promotion of low-calorie products as an ‘*outdated*’, and harmfully (e.g. ‘*toxic*’, ‘*messed up*’) stereotypical and sexist tactic used to target (‘*I don’t think they would make it as a marketing argument for their drink towards men*’, Sarah, white, 20s heterosexual, drinker) and encourage alcohol use among women (‘*it’s like you won’t get fat but you will get pissed*’, Vicky, White, 30s, heterosexual, ex-drinker). It was regarded as feeding into, and reproducing, ‘*diet and thin culture*’ and patriarchal standards of beauty that reduce women to objects of appearance, and as playing on their anxieties around weight and appearance, for commercial purposes. Most expressed annoyance and anger towards the use of calories as a marketing strategy, felt that it was an example of female-targeted marketing that was ‘*weirdly based on aesthetics*’ (Erin, White, white, heterosexual, drinker), and suggested they would avoid such brands and products as a result. For example, Gemma (White, 20s, LGBTQ+, drinker) explained how ‘*it just fucking annoys…it’s capitalism. They [brands] want us to feel insecure so they can make money*’. Similarly, Fiona (White, heterosexual, 30, drinker) felt that brands were ‘*selling you the problem and the solution in one. The problem is alcohol has got a lot of calories, but don’t worry because this alcohol has got less calories*’. Kat (White, 30s, LGBTQ+, 30s) went further to associate this focus on appearance with the demands placed on women when participating in nightlife drinking environments, suggesting that a focus on calories feeds into ‘*the need to be skinny to go to a [night] club*’. Thus, a focus on calories was felt to potentially impact well-being through reinforcing negative body image, and contradicted women’s aims of body positivity. For instance, Bianca (Black, 20s, heterosexual, drinker) expressed how such messaging was damaging to women’s ‘*confidence*… *mak[ing] them think “Do I need the lower calorie one like?” [and] affecting your mental health*’.

For some, this annoyance and critique extended to ACLs as a public health policy to address obesity and alcohol use, and was discussed as using one health issue, (i.e. anxiety over body image) to address others (e.g. weight, alcohol consumption), in ways that may exacerbate the negative impact of body image concerns on mental health. For example, Samantha linked calorie labelling to marketing, describing a focus on calories as ‘*incredibly toxic*’ and a ‘*cop out*’ with regard to marketing regulation. This suggests a degree of cynicism towards such strategies, with women prioritizing maintaining healthy body image, over weight control and their alcohol use. For example, Tasha (White, 20s, heterosexual, drinker) described calorie marketing as ‘*playing into the fact of people calorie counting and watching their weight, but not really promoting healthy body image or confidence*’ and extended this critique to calorie labelling, which she felt would be ineffective other than triggering ‘*people worrying about how much they’re drinking because of the weight they might put on*’. She also associated calorie information with reducing the pleasures involved in a night out drinking through inducing ‘*guilt*’, stating that ‘*realistically, if you’re out on a night out, you’re gonna stand there and look at how many calories are in it, and that’s not going to make you feel great*’. Thus, calorie information was rejected by many, and these negative attitudes towards calorie marketing through a feminist anti-diet and body positivity discourse extended to views on ACLs, which may potentially impact the effectiveness of the policy among some women.

### ‘*You can go out and still be able to enjoy yourself and not have to limit your alcohol consumption*’: potential impact of calorie marketing, and ACLs, on alcohol practices

Some women supported ACLs as a useful health response and compared it to mandatory calorie labelling on food and soft drinks (‘*I mean I don’t disagree with it, ‘cause we have it on soft drinks, so yeah, why not?*’; Nicole, White, 20s, heterosexual, drinker). For example, for Nicole, calorie labelling was regarded as a ‘*standardised*’ way of ‘*making people more aware of reducing their alcohol consumption, and how alcohol feeds into calories*’. However, whilst expressing that she did not view ACLs as being targeted at women specifically, she did state that if this was the case, she would no longer support the measure (‘*If it was actively sort of targeting like girls, you know that would be very problematic*’). Despite ACLs being a population-level approach, if viewed as an extension of brand marketing that emphasizes low-calorie content of products to target women, it may thus be rejected. As shown in extract one below from an ex-drinker, some viewed calorie labelling as a potentially more effective approach than mandatory unit labelling (i.e. labelling stating the number of ‘units’ in alcoholic drinks, 1 unit = 10 ml or 8 g of pure alcohol). In contrast, others highlighted the ineffectiveness of unit information, and were more sceptical of the relative benefits of calorie labelling. For example, Alice (White, 20s, heterosexual, drinker) questioned the usefulness of calorie labelling stating that ‘*if units don’t work, then I don’t really see what calories is gonna add to that apart from people worrying about how much they’re drinking because of the weight they might put on*’. She perceived both approaches as ineffective, and as discussed, framed the provision of calorie information as generating anxiety over alcohol use and weight, without changing actual behaviour.

*Extract 1*

*I still to this day don’t massively understand what a unit of alcohol looks like…but I understand enough about calories, like how much I’m meant to have a day, what’s bad in terms of calories, and I think from a harm reduction perspective [mandatory calorie labelling] has potential to make a difference with some people*.

(Lisa, White, 30s, heterosexual, ex-drinker)

A number of potential changes to alcohol consumption practices as an outcome of increased awareness of calorie content through both marketing and calorie labelling were discussed. However, participants tended to speak in the third person and discussed impact on the behaviour of others, but not their own. A small number of participants felt that increased awareness of the relatively lower calorie content of some drinks would result in the consumption of lower calorie options. This was discussed as having the potential positive impact of reducing calorie intake and weight loss, but failing to reduce alcohol intake, in that lower calorie options allowed consumers to ‘*enjoy’* drinking the same amount of alcohol whilst reducing calories. For instance, Kerry (White, 30s, heterosexual, ex-drinker) felt that an awareness of calorie content through marketing and labelling was ‘*good if you’re like looking after your health. If you’re trying to lose a bit of weight, if you’re on Slimming World and that fits into your points, then it’s good that you can go out and still be able to enjoy yourself and not have to limit your alcohol consumption*’. A discourse of pleasure was at play here, with the role of calorie content as aiding lower calorie choices, being viewed favourably by allowing for enjoyment through drinking and intoxication (with female friends), while also inducing weight loss or preventing weight gain. Michelle (White, 20s, heterosexual, drinker) also referred to an awareness of low-calorie options as ‘*allowing you to have a good time and have a drink, whilst staying on this so-called “diet” you’re on, but obviously, it can be harmful*’. When asked to clarify what the harm may be, she drew on both the anti-diet and pleasure discourses previously discussed, explaining that it ‘*mixes alcohol and night-life with diet culture. It just doesn’t sit right…when you’re going on a night out, go and have fun, don’t worry about your weight or your appearance*’. Such statements support the intended aim of ACLs in impacting an individual’s calorie intake, but not in reducing overall alcohol use, with drinking and intoxication being prioritized through a discourse of pleasure. Importantly, those who discussed ACLs positively and expressed appeal for low-calorie products, were those who reported they were already aware of the calorie content of alcohol drinks. They discussed existing strategies to reduce the impact of the calories consumed through alcohol on weight gain [e.g. drinking low-calorie options (spirits and soda, reduced calorie mixers)] and as such felt that people employing these strategies may not require calorie information. Instead, they would continue to employ these pre-existing strategies, as opposed to switching drinks or reducing their drinking as a result of the provision of calorie information. For example, Amy (White, 20s, LGBTQ+, drinker) discussed how low-calorie alcohol ‘*would appeal to me, but I wouldn’t buy anything like that* [product promoting lower calories], *‘cause if I was on a night out I would drink like a double vodka, lime and soda, cause I know it’s really low in calorie*’. In the same manner, Kerry (White, 30s, heterosexual, ex-drinker) felt consumers that may find calorie information useful were those already ‘*counting the calories*’, researching the calorie content of alcoholic drinks, and choosing drinks such as Gin and Tonics that would not ‘*be way off the scales*’.

A generational distinction was made when suggesting who would be more inclined to drink lower calorie products and act upon calorie labelling, with participants describing older women, such as those who were mothers, and women in early midlife, as being most susceptible. For example, Danielle (Black, 20s, LGTQ+, drinker) did not consider calories in relation to her own alcohol use and diet but described how calorie marketing and labelling ‘*screamed Mum vibes*’, and how her ‘*mum and grandma almost exclusively only drink gin because it’s the healthy alcohol, it’s the one with the least amount of calories*’. Here she made associations between low-calorie content and health, describing gin as relatively ‘*healthy*’. Similarly, Zoe (Black, 20s, LGBTQ+, drinker) felt that calorie labelling was a ‘*good idea*’ but was more relevant to middle-aged women who she regarded as more conscious of calories, rather than younger women like her and her friends who had ‘*higher metabolisms*’. Recalling how ‘*any women in my life that are 40s plus, are all about being a bit healthier*’, she also associated lower calories with health. This suggests a potential unintended consequence of calorie information provision, with associations between alcohol and health potentially leading to consumers interpreting lower calorie alcoholic drinks as healthier options, in ways that overlook the negative health impacts of alcohol itself. Ex-drinkers in particular expressed concern over associations between alcohol and health, such as Maya (White, 30s, heterosexual), who felt that low-calorie options were ‘*misleading because it’s saying that it’s healthy, but it’s not, like alcohol is not healthy*’. She went onto describe calorie information as ‘*harmful and outdated [and] really ironic because of the harm that alcohol is doing to you, but like, “Oh no, but don’t worry, like it’s only 50 calories”, you know? I mean, it’s actually laughable*’.

The impact of calorie information on problem use was discussed by those currently sober due to previous drinking problems. As shown in extract three below, one participant, Jenny, stated that being informed of the calorie content of alcoholic products, would have potentially led to her drinking less alcohol alone within the home, but would not influence her drinking with friends in the nighttime environment. She went on to suggest that if her drinking had gotten more problematic, calorie labelling would have led to her drinking stronger alcohol such as spirits due to their lower calorie content—‘*if I’d got to the point where I quote unquote “needed a drink”, would have then gone to something stronger and ultimately more dangerous*’.

*Extract 3*

*I think it would have prevented me from doing things like having one or two cans of cider on a night, it wouldn’t have stopped me from going out and having like a massive binge drink, but it potentially would have curbed some of the more day-to-day habitual drinking.*

(Jenny, White, 20s, heterosexual, ex-drinker)

An awareness of the calorie content through marketing and labelling was thus felt to have potential negative unintended consequences among heavier and problematic drinkers, through switching to ‘s*tronger*’ drinks like spirits, which when drank with low-calorie mixers or straight, were believed to contain less calories. Spirit drinking was viewed as indicative of an enhancing problematic relationship with alcohol, which was echoed by other participants who were currently sober due to self-defined problematic use. Like Jenny, they expressed concern that an awareness of calorie content could have led them, and others, to spirit consumption, which could result in an increase in the total amount of alcohol consumed. For example, Sally (White, 30s, heterosexual, ex-drinker) felt that ACLs ‘*might push someone into, drinking hard liquor, because thinking about my own [drinking] that was really where I was at, it was ‘well, I’ll just drink straight vodka*’. Similarly, Laura (White, 30s, heterosexual, ex-drinker) extended this thought process to all drinkers, suggesting that ‘*because spirits have less calories, everyone will like get on the harder stuff….I mean we all know that wine has a killer amount of calories so just get on the gin with the zero tonic. I don’t think it’s gonna help is my answer to that question*’. Current drinkers also agreed, suggesting that rather than drinking less as an outcome of calorie awareness, people who ‘*want to get drunk*’ would employ other strategies ‘*to cut the calories down, like not drink wine or not have a mixer and just drink straight vodka, so it could kind of go the other way*’ (Sarah, White, 20s, heterosexual, drinker).

In contrast, some discussed how an awareness of the high calorific content of wine would have failed to impact their previous (problematic) drinking practices (‘*I always drank wine and that was probably the most calorific, it didn’t impact me personally*’; Tammy, White, 30, heterosexual, ex-drinker), as wine consumption had better met their desired level of intoxication (‘*it got me the most drunk off quick*’). Again, here we see how intoxication is prioritized over reducing calorie intake, but in this case for the purposes of self-medication, rather than pleasure. Relatedly, ACLs were viewed as potentially ineffective within the context of drinking to intoxication, with intoxication itself felt to impede understanding and concern over the calorie intake at the time of drinking. For example, Vicky (White, 30s, heterosexual, ex-drinker), expressed that calorie information may be useful ‘*until a point where they’re not really caring about calories anymore, because they’re too drunk to care*’. Laura agreed when stating that ‘*Let’s be honest. If you’re out on a Friday night, after a few, are you then gonna start checking the bottles and the labels, n*o’.

### ‘*I don’t care about calories’*: potential impact on dietary/eating practices

Many women expressed a general lack of consideration for calories and in turn dismissed calorie marketing and ACLs, stating that they ‘*don’t care about calories*’. Alcohol was framed as a treat within their overall diets, and exposure to calories was criticized for inducing feelings of guilt, and as discussed, as impacting on the fun gained from alcohol consumption and intoxication through a discourse of pleasure. As Fiona (White, heterosexual, 30, drinker) explained, ‘*it does annoy me because I shouldn’t feel guilty about wanting to have a drink and then think “Oh, let’s see how many calories this has got”. Like I’m not interested I just want to go out and have a good time with my friends*’. Overall, most participants described the purpose of a night out drinking as being ‘*carefre*e’, ‘*having a good time*’, and not ‘*watching [their] weight*’ (Michelle, White, 20s, heterosexual, drinker). Bianca (Black, 20s, heterosexual, drinker) agreed that drinking on a night out was about being ‘*carefree*’, but felt that low-calorie options would aid this carefree attitude, in that ‘*if*’ she was ‘*bothered about weight or appearance, etc., or just wanted to keep the calories low and I was going somewhere, I might be like, “Oh yes, I can drink this beer. I’m fine,” and it would make me carefree*’. Whilst these women rejected diet culture themselves and disclosed that they did not count calories, they did however feel that an awareness of low calories would allow women to continue to enjoy alcohol use, whilst lowering calorie intake. A discourse of pleasure was thus again at play in accounts of low-calorie options and the potential impact of ACLs on consumer behaviour, with enjoyment and fun (‘*good time*’, ‘*carefree*’) being prioritized over changing eating practices. Among some, pleasure was again entwined with the anti-diet rhetoric previously discussed, with criticism of inducing ‘*guilt*’ discussed as impeding enjoyment.

Calorie marketing and low-calorie products were also rejected on the assumption that they would compromise on taste, reinforcing usual consumption practices and ‘*drinking what you like*’. For example, Paige (White, 20s, heterosexual, drinker) stated that ‘*if a beverage tasted nice and it was a lot more calories, then I’d go for taste over calorie intake*’. Similarly, Summer (Black, heterosexual, 20s, drinker) criticized a friend on the basis of taste describing how for health and fitness reasons she ‘*will only drink vodka and tonic… it’s absolutely disgusting. I don’t know why you’d put yourself through it*’, suggesting her friend should ‘*just gain a few pounds*’. This suggests that some female consumers would dismiss the labelling of alcoholic drinks as calorific, prioritizing taste, as a form of pleasure, within their diets over calorie intake. However, some expressed more concern over the high sugar content of products and drinks (e.g. Pink gin, cocktails), discussing avoiding drinks high in sugar for health reasons and on the basis of taste ‘*it’s “low calorie” but so high in sugar. Why would I want to ingest this*?’ (Suzy, Black, 20s, heterosexual, drinker).

Calorie awareness through marketing and labelling did appeal to some as a diet aid, for example Rachel (White, 20s, heterosexual, drinker), who expressed that ‘*lower calories definitely appeal to girls. I know it would for me*’. They were also viewed as ‘*lighter*’ options that prevented ‘*bloating*’, and as such had physical and aesthetic benefits. Discussion surrounded the existing dietary strategies employed to manage excess calorie intake, including adapting diets by ‘*eating better*’ or ‘*healthier*’ during periods of alcohol use to accommodate for the calories consumed through alcohol use, or exercising more to maintain an equilibrium. Sarah (White, 20s heterosexual) stated that she knew people who when drinking was ‘*wary of either how many times they drink in the week, how many drinks they’ve had, and compare[d] that and balanced it out with the gym or the food they eat that week*’, for example, ‘*not getting a take away that night, eating healthy, or going the gym another week*’. This suggests that for some, increased calorie awareness may result in healthier eating or exercising more to offset the excess calories consumed through alcohol, thus preventing weight gain and meeting the intended aim of ACLs of reducing calorie intake as a strategy to address obesity.

Unintended negative consequences on eating patterns were also envisaged, such as eating less to free up calories for alcohol consumption, a practice some had engaged in themselves. Samantha (White, 40s, heterosexual), an ex-drinker, recalled how this practice of ‘*drunkorexia … was definitely part of my story. I would save my calories for wine, like I wouldn’t have a dessert, I would have another glass of wine, and I see that with my friends and different people, sometimes I wouldn’t eat dinner*’. Stephanie (British-Indian, 30s, heterosexual), another ex-drinker, agreed that ACLs would ‘*just give people numbers to work with because what people do if people are generally worried about calories in alcohol, they just cut back on what they’re eating*’. As shown in extract four below, from an interview with Ava, there was particular criticism from participants with a history of eating disorders on the impact of calorie marketing and ACLs on diet and eating patterns. It was felt that both were ‘*unhelpful*’ in reinforcing unhealthy thought patterns and eating behaviours that were detrimental to physical and mental health. As well as discussing the impact calorie marketing and labelling would have on her attitudes to eating and behaviour, Ava also drew on the pleasure discourse, discussing how an awareness of calories in alcohol would ‘*put a dampener on*’ her night out.

*Extract 4*

*It’s [marketing and ACLs] making it seem as though there’s a good choice and the bad choice. It’s easier when that’s not there, because if it’s there, I’m going to check, that’s just what my brain’s gonna do. It’s automatic and I don’t want to be doing that. It’s like when they were moving towards making it mandatory to have like the calorie content on like menus, because of my history, if there’s the option that it’s lower, I’m probably gonna go for it just because unfortunately*.

(Ava, White, 19, LGBTQ+, drinker)

## DISCUSSION

The article presents findings of qualitative research exploring women’s attitudes towards calorie messaging as an alcohol marketing strategy, and calorie labelling as a public health policy, in the UK. In 2020, the UK Government (DCSH, 2020) announced an intention to consult on introducing standardized ACL, but no progress has been made to date. In this discussion, we focus on the implications the research has for ACLs as a health policy to address alcohol use and obesity.

First, the implementation and effectiveness of ACLs must be considered within the existing commercial environment in which some alcohol brands are promoted through calorie-based marketing. ALCs may thus be undermined by existing marketing messages that use calorie references to *encourage* sales and use. We found that the consumption of low-calorie products may assist with the calorie-reducing aims of ACLs among some calorie-conscious consumers (Robinson *et al*., 2021b). Eating healthier in the days surrounding alcohol use or engaging in physical activity to compensate for the calories consumed from alcohol was also discussed, which suggests that increasing awareness of the calorie content of alcohol can promote weight management (Robinson *et al*., 2021b). However, lower calorie products and marketing, and labelling, were perceived as appealing to older women for these purposes. There was very little evidence that consumers would reduce their alcohol intake due to increased awareness of calorie content, either as a result of marketing messages, or ACLs.

Second, whilst research has shown support for ACL (Gibson and Shirreffs, 2013; Maynard *et al*., 2018; AHA, 2021a; IAS, 2021), this research found that many women were critical of calorie marketing through a contemporary feminist anti-diet culture lens, with some interpreting calorie information as an extension of the diet industry that draws on women’s weight anxiety for commercial gain. For some, this critique extended to ACLs which they felt created the same sense of guilt among women in relation to weight and appearance. Associations between ACLs and brand marketing, and critiques of calorie information through a body positivity and anti-diet discourse, may lead to female consumers rejecting ACLs, thus limiting its effectiveness. Future research is needed that considers and measures the gendered impact of ACLs within the broader context of commercial determinants, as well as a consideration of body positivity discourse that forms a key feature of mainstream contemporary feminism (Johnson and Taylor, 2008; Sastre, 2014; Darwin and Miller, 2021).

Third, a discourse of pleasure when discussing calorie information was at play, and for some, lower calorie options were viewed favourably by allowing consumers to continue to enjoy the pleasures associated with alcohol use (i.e. fun with friends, which is also promoted in marketing, Atkinson *et al.*, 2022a), whilst also allowing them to reduce calorie intake. The context of consumption also appeared to be important in gendered ways, with calorie information being perceived as limiting the fun associated with a ‘night out’ drinking in venues with female friends, and intoxication itself impeding an understanding and concern calorie for at the time of drinking. This pleasure discourse was entwined with an anti-diet rhetoric, with calorie information being viewed as impacting pleasure, by reproducing the ‘guilt’ induced by diet culture and exacerbating body image concerns.

Next, it is vital that the potential unintended consequences of ACLs are considered. Whilst ACLs have ‘good intentions’ to improve health, evidence of its long-term effectiveness and acceptability is limited and it may produce unintended adverse effects (Robinson *et al*., 2021a). This includes consumers (with problem drinking) seeking out higher strength drinks with fewer calories per ABV (e.g. spirits) and research should consider the impact on people with differing drinking practices and on the spectrum of problem use. Compensatory behaviours that increase the likelihood of experiencing alcohol-related harm such as restricting food intake immediately prior to or after drinking may also be encouraged among some (Maynard *et al*., 2018; Robinson *et al*., 221b). Labelled ‘drunkorexia’ (Barry and Piazza-Gardner, 2012; Eisenberg and Fitz, 2014), this practice may be more prevalent among consumers with a history of eating disorders, and the impacts of calorie labelling on these individuals mental and physical health was a concern to participants.

Last, with a lack of health warning and energy content information on labels, the use of calorie-based marketing to encourage sales and use, has the potential to mislead consumers by framing products as healthy and detracting from the harm associated with alcohol consumption (Haynes *et al*., 2022; Cao *et al*., 2023). In the UK, alcohol marketing is regulated through a reactive complaints-led system of self-regulation by the alcohol and advertising industries [e.g. represented through the Portman Group and Advertising Standards Authority (ASA)], and co-regulation with the Office of Communications (Ofcom). Self-regulation is critiqued as a deliberate attempt to delay government regulation and proactive monitoring, and as ineffective in preventing breaches of codes that aim to regulate the nature of marketing by prohibiting particular content (Kier and Stafford, 2019). In relation to health-related claims, in the UK alcohol producers and marketers ‘*may give factual information about product contents, including comparisons, but must not make any health, fitness or weight-control claims*’. They are allowed to make claims of ‘*low-alcohol*’, ‘*reduced alcohol*’ and ‘*reduced energy*’ (ASA, 2023). The Portman Group’s (2022a) voluntary guidance also states that ‘*drinks with more than 1.2% ABV can give factual information about calorie content, such as “90 calories”, but should avoid using phrases like ‘only 90 calories*’. However, there are various examples of complaints for breaches of these (e.g. ASA, 2021a,b), suggesting the current system is ineffective. This feeds into wider debates around the need for more comprehensive marketing restrictions (Boniface *et al*., 2021).

Policy should also consider how best to prevent ALCs leading to alcohol products being interpreted as relatively ‘*healthy*’ in ways that overlook and disguise the potential for alcohol-related harm. For example, Pitt *et al*. (2023) conducted an online qualitatively survey of 497 Australian women who had consumed alcohol in the last year and found that ‘low-calorie’ or ‘low-sugar’ products were perceived as providing healthier alternatives to traditional alcohol products and aligned with women’s concerns over weight, yet some expressed concern that their availability increased alcohol consumption by reducing the perceptions of risk associated with alcohol. It could also be argued that standardized nutrition declarations on alcohol including calorie information, could further normalize alcohol as a relatively benign product by further positioning perceptions of alcohol as a food product versus a psychoactive addictive substance. Recent experimental research (Hobin *et al*., in press) found that consumers exposed to alcohol containers with nutrition marketing (including calories), were more likely to report intentions to try and buy the product compared to consumers exposed to containers without nutrition marketing. Moreover, consumers exposed to nutrition marketing compared to a health warning label (e.g. the risk of cancer) reported intentions to consume more of the product. Thus, calorie labelling, without health warnings, may backfire and motivate consumers to consume more because they believe it is healthier/less harmful. Calorie labelling should be presented alongside labels that highlight the potential health harms of alcohol use such as the risk of cancers, a combined approach that will come into effect as mandatory in Ireland in 2026 (Maynard *et al*., 2018; Carroll, 2023; Zhong and Rahman, 2023).

While the article considers calorie-based marketing and labelling in terms of its effectiveness or non-effectiveness in changing drinking and eating behaviours, the nutritional labelling of drinks and food items may also be considered from an ethical and human rights perspective (Alcohol Focus, 2022; Alcohol Health Alliance, 2022). This asserts that individual consumers have the right to be provided with the information needed to make informed choices, even if their choice is to subsequently ignore calorie content. From this perspective, information should be provided even in the absence of evidence that it contributes to changes in consumption. However, as discussed, consideration of unintended and adverse consequences that could lead to counterproductive outcomes is important. It could similarly be argued that consumers have the right to be protected from the adverse impacts of health promotion such as labelling, and the negative impact alcohol marketing that uses calorie information may have on health. Indeed, the Royal Society of Public Health (2020), shifted its position on calorie labelling to address obesity, raising concerns over its impact on the mental health of those with eating disorders. Instead, they call for changes to the environment in which ‘healthy’ and ‘unhealthy’ food and drink options are promoted and made available. Such factors are an important consideration for future research and policy evaluation.

## CONCLUSION

Mandatory labelling of alcoholic drinks with calorie information may form one component of a strategy to reduce alcohol harm and obesity, alongside other policies such as a taxation and price regulation and regulating availability and marketing. This research highlights that ACLs should be considered within the wider commercial context of alcohol marketing that draws on calories to promote sales and consumption, consideration of the gendered factors that may lead some to rejecting ACLs, and the potential for unintended consequences. A number of limitations of the research should be acknowledged and include its qualitative nature which limits the generalisability of the results, particularly to men and other genders. The research was conducted in the UK and may not be transferable to other countries. Despite this, the article makes a valuable contribution in providing in-depth qualitative insight into women’s views and attitudes to calorie-based marketing, and ACLs. Further UK and international qualitative research is needed with a more diverse sample of participants, to provide insight into how calorie information is considered in the everyday lives of consumers, and how it impacts their alcohol use and drinking patterns.
