# Correction to: Qualitative analysis of UK women’s attitudes to calorie-based alcohol marketing and alcohol calorie labelling

**DOI:** 10.1093/heapro/daae029

**Published:** 2024-03-21

This is a correction to: Amanda M Atkinson, Beth R Meadows, Erin Hobin, Lana Mae Vanderlee, Harry Sumnall, Qualitative analysis of UK women’s attitudes to calorie-based alcohol marketing and alcohol calorie labelling, Health Promotion International, Volume 39, Issue 1, February 2024, daae006, https://doi.org/10.1093/heapro/daae006

The originally published version of this article omitted details of the cited paper Hobin *et al.*, in press, in the Discussion section and reference list. The article has been updated to add these details.

